# Case Report: Albumin combined with plasma exchange in the treatment of refractory thrombotic thrombocytopenic purpura

**DOI:** 10.3389/fmed.2026.1843101

**Published:** 2026-07-14

**Authors:** Yi Zhao, Linman Cao, Rongrong Wang, Linzheng Luo, Yijing Liu, Mingyu Li, Boci Li, Bowen Ren, Chun Zuo, Bao Chu, Mingmin Zhao, Lei Zhao, Xing Xing, Na Li

**Affiliations:** 1 Graduate School, Hebei North University, Zhangjiakou, Hebei, China; 2 Hebei Provincial People’s Hospital, Shijiazhuang, Hebei, China; 3 Graduate School, Hebei Medical University, Shijiazhuang, Hebei, China; 4 Graduate School, North China University of Science and Technology, Tangshan, Hebei, China; 5 Cangzhou People’s Hospital, Cangzhou, Hebei, China

**Keywords:** ADAMTS13, cerebral infarction, plasma exchange, therapeutic plasma exchange, thrombotic thrombocytopenic purpura

## Abstract

**Objective:**

This study aims to analyze the clinical characteristics and causes of misdiagnosis in patients with thrombotic thrombocytopenic purpura (TTP) presenting with initial neurological symptoms, so as to provide reference for reducing misdiagnosis; to explore the feasibility and efficacy of albumin combined with plasma exchange in the treatment of TTP patients when plasma inventory is insufficient; and to put forward key precautions for TTP diagnosis and treatment, so as to provide strategic support for the treatment of similar cases.

**Methods:**

Clinical data of the patient (medical history, examination results, diagnosis and treatment records, etc.) were collected retrospectively; albumin combined with plasma exchange was adopted when plasma inventory was insufficient; laboratory indicators and symptom recovery were dynamically monitored to evaluate the effectiveness and safety of the treatment; misdiagnosis risks and the current status of alternative treatment were analyzed combined with literature review.

**Conclusion:**

Thrombotic thrombocytopenic purpura with initial neurological symptoms is easily confused with cerebral infarction, and thrombocytopenia of unknown origin is a key clue for differential screening. TTP should be excluded before platelet transfusion; when plasma inventory is insufficient, albumin combined with plasma exchange is feasible and effective for the treatment of AB-blood-type TTP, which can provide an alternative strategy for clinical practice; early diagnosis, timely targeted treatment and multidisciplinary collaboration are the keys to improving the prognosis of TTP patients.

## Introduction

Thrombotic thrombocytopenic purpura (TTP) is a rare and potentially life-threatening thrombotic microangiopathy. It is characterized predominantly by acute onset, frequent neurological symptoms, concurrent involvement of multiple systems, and an extremely high mortality rate if treatment is delayed. Therapeutic Plasma Exchange (TPE) is conventionally the first-line treatment for TTP. The recommended volume of plasma for a single TPE session is 2000–3000 milliliters. Special blood type plasma, such as AB plasma, is often in short supply, which makes it extremely challenging to source compatible plasma for patients requiring daily TPE sessions. This paper reports a case of TTP presenting with initial neurological symptoms. The patient was misdiagnosed with cerebral infarction at the first consultation, and the condition continued to deteriorate after conventional symptomatic treatment. A definitive diagnosis of TTP was confirmed subsequently, and the patient achieved clinical remission after standardized treatment. In the present case, an AB-positive (ABO type AB, RhD-positive) patient achieved favorable therapeutic outcomes via a treatment regimen combining albumin and plasma exchange when sufficient clinical plasma inventory was unavailable. This case report provides two key clinical implications: first, for patients presenting with initial neurological symptoms complicated by multiple system damage and unexplained thrombocytopenia, routine screening for microthrombotic diseases such as TTP is required prior to platelet transfusion; second, this regimen provides a feasible clinical strategy for alternative plasma exchange therapy for TTP patients when plasma inventory is in shortage.

The patient is a 64-year-old female who presented with acute onset of neurological symptoms predominantly characterized by numbness in the right hand and stiffening of the tongue root 5 days prior to admission to our hospital. Upon initial presentation at the first hospital, a complete cranial computed tomography (CT) scan revealed no obvious abnormalities, and cranial magnetic resonance imaging (MRI) showed no acute lesions. A tentative diagnosis of cerebral infarction was made, and antiplatelet aggregation therapy was administered, yet the patient’s symptoms were not relieved. A routine blood test showed a platelet count of 13 × 10^9^/L and hemoglobin (HGB) was 70 g/L (One month prior to this admission, both PLT and HGB were within normal ranges on physical examination). Platelet transfusion therapy was subsequently given. Afterward, the patient’s consciousness level declined to coma, accompanied by convulsions and fever, with progressive decreases in PLT. The patient was therefore transferred to the Department of Neurology of our hospital. There was no significant past medical history, personal history, or family history.

Physical Examination on Admission: The patient presented with coma and was receiving mechanical ventilation via endotracheal intubation. Scattered ecchymoses that do not blanch on compression were observed on the extremities and abdomen. No obvious abnormalities were detected in the cardiopulmonary and abdominal examinations, and no edema was found in the extremities. The patient had hematuria. Neurological Examination: Bilateral pupils were equal, round and normally sized, with a diameter of 3.0 mm. The pupillary light reflex was sluggish, and bilateral Babinski signs were positive. No other positive neurological signs were identified. NIHSS score: 26 points.

The laboratory tests upon admission (see Table 1) indicated severe thrombocytopenia, severe anemia, impaired hepatic and renal function, abnormal myocardial zymogram, and active systemic inflammatory response.

**TABLE 1:** First major laboratory test results during hospitalization.

Test Items	Test results	Reference range
PLT (10^9/L)	10.0	125.0–350.0
HGB (g/L)	65.0	115.0–150.0
WBC (10^9/L)	11.8	3.5–9.5
CRP (mg/L)	34.2	0.0–10.0
PCT (ng/mL)	0.5	<0.06
SAA (mg/L)	543.3	0–10.0
Percentage of schistocytes (%)	4.0	–
Reticulocyte percentage (%)	–	0.5–1.5
Prothrombin Time(s)	13.4	9.8–12.1
Fibrinogen (g/L)	2.0	45692.0
D-dimer (mg/L FEU)	14.0	0–0.5
LDH (U/L)	2371.9	120.0–250.0
CK (IU/L)	137.2	40.0–200.0
CKMB (IU/L)	61.3	0.0–24.0
hs-cTnT (pg/ml)	213.7	<70.0
Total bilirubin (μmol/L)	53.7	0.0–23.0
Direct Bilirubin (μmol/L)	11.8	0.0–6.8
Alanine aminotransferase (U/L)	278.2	7.0–40.0
Aspartate aminotransferase (U/L)	330.3	13.0–35.0
Urea (mmol/L)	10.3	3.1–8.8
Creatinine (mmol/L)	93.0	41.0–81.0
GFR (ml/min)	56.1	–
Urinary occult blood	+++	–
Anti-histone antibody (AU/mL)	27.5	<20.0
Antimitochondrial antibodyM2 (AU/mL)	39.9	<20.0
Immunoglobulin G (g/L)	18.1	8.6–17.4
Immunoglobulin A (g/L)	0.8	1.0–4.2
Immunoglobulin M (g/L)	0.5	0.5–2.8
ESR (mm/h)	>140	–
Anti-Ro-52 (AU/mL)	39.1	<20.0
Complement C3 (g/L)	0.8	0.7–1.4
Complement C4 (g/L)	0.3	0.1–0.4
Complement C1q(mg/L)	127.2	159.0–233.0
Coombs test	Negative	–
ADAMTS13 activity	0.62%	40%–130%
ADAMTS13 inhibitory antibody	60.66 U/mL (positive)	0–10 U/mL

**FIGURE 1:**
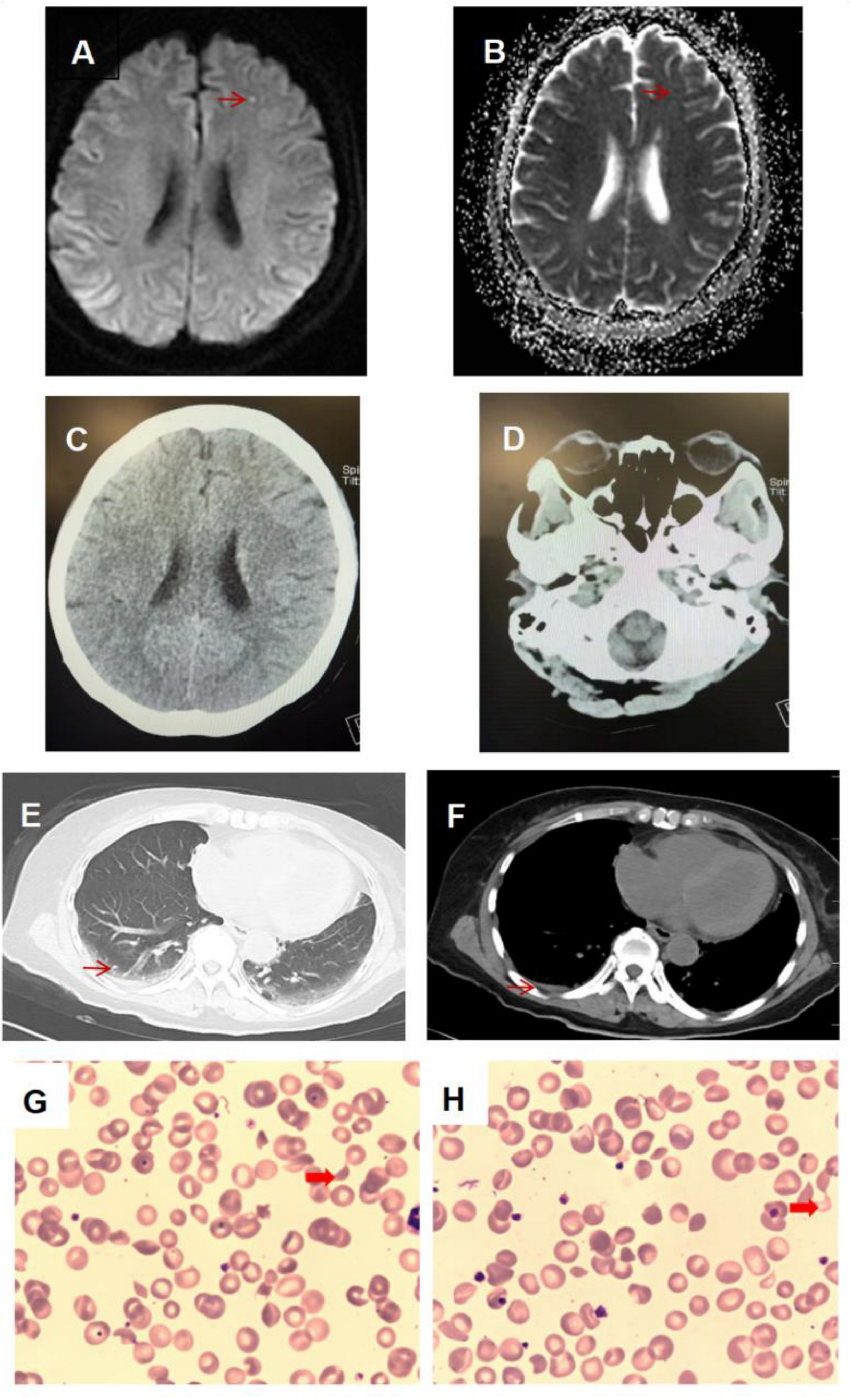
Imaging examinations of the patient on admission. **(A)** DWI image of brain MRI on admission; **(B)** ADC image of brain MRI on admission; **(C,D)** brain CT on admission; **(E,F)** chest CT on admission; **(G,H)** microscopic field of view of schistocytes.

Admission imaging examinations (see Figure 1): Cerebral DWI: lacunar cerebral infarction in the left frontal lobe; Cerebral CT: no obvious abnormality detected; Chest CT: a small amount of inflammation in both lungs. Electroencephalogram: 1. Epileptiform discharges in the right temporal-parietal region. 2. Generalized slow waves in all leads.

According to the localization principles of neurology, a positive bilateral Babinski sign is localized to the bilateral corticospinal tracts; disturbance of consciousness is localized to either the bilateral cerebral cortex or the ascending reticular activating system; epileptic seizures are localized to the cerebral cortex in the right temporal region. Following the MIDNIGHTS diagnostic principle, this patient is an elderly female presenting with acute onset and progressive worsening of symptoms. No abnormal blood pressure or hypoglycemia was observed at disease onset, and there is no history of toxic exposure or trauma. The qualitative diagnosis suggests etiologies related to stroke, infection, inflammation and neoplasm. Preliminary diagnosis: Unconsciousness (to be investigated), etc.

In the absence of cerebrovascular imaging evidence for this patient, the stroke cannot be classified via the conventional TOAST typing system. Furthermore, the current imaging findings are inconsistent with the patient’s severe clinical symptoms and signs, thus further etiological investigation is required. Based on the preliminary localization and qualitative diagnosis, relevant laboratory examinations were completed (see Table 1). Integrated analysis of the results showed progressive anemia, elevated reticulocyte count, hyperbilirubinemia dominated by indirect bilirubin, increased lactate dehydrogenase level, and 4% red blood cell schistocytes observed under microscopic examination of peripheral blood smears (Figure 1). Hemolytic anemia was thus considered, and the Coombs test result was negative. Microangiopathic Hemolytic Anemia (MAHA) was considered highly probable. MAHA mainly includes disseminated intravascular coagulation (DIC), TTP, Hemolytic Uremic Syndrome (HUS), etc. The patient’s score on the Chinese DIC Diagnosis Scoring System (CDSS) was 5 points, indicating low probability of DIC. The patient concurrently presented with MAHA, neurological symptoms, renal dysfunction, fever and severe thrombocytopenia, which corresponds to the “TTP pentad”. Furthermore, the patient’s PLASMIC score for TTP risk stratification was 6 points, leading to high suspicion of TTP. Based on current clinical manifestations, the diagnosis is more inclined to TTP, although HUS still cannot be ruled out. Timely submission of blood samples for ADAMTS13 detection.

**FIGURE 2:**
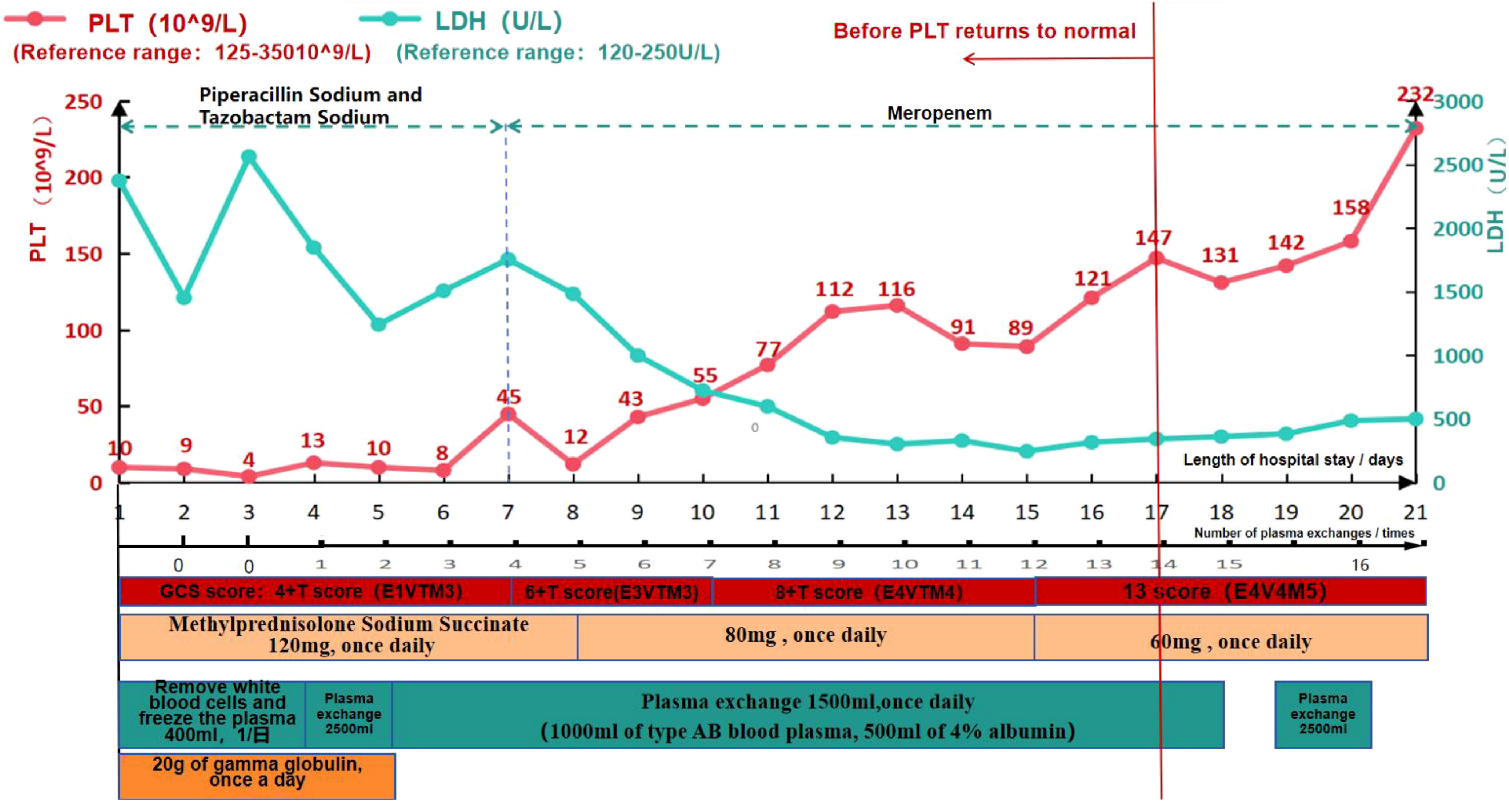
Dynamic trends of platelet count, lactate dehydrogenase level and corresponding sequential therapeutic interventions during hospitalization. The horizontal axis represents hospital stay days and the number of plasma exchange sessions; the left vertical axis indicates PLT count (reference range: 125–350 × 10^9^/L), and the right vertical axis shows LDH activity (reference range: 120–250 U/L). The red curve denotes PLT, and the teal curve denotes LDH. Anti-infective agents, daily dosage of methylprednisolone sodium succinate, intravenous gamma globulin regimen, therapeutic plasma exchange protocols (combination of type AB plasma and 4% albumin) and serial GCS-T scores are labeled chronologically. The vertical red line marks the critical time point before PLT returned to normal, illustrating that plasma exchange was continuously administered prior to platelet recovery.

In terms of treatment, when TTP was highly suspected, we initiated therapy with gamma globulin, corticosteroids and plasma transfusion without waiting for the results of laboratory tests. According to the Clinical Practice Guidelines for Diagnosis and Treatment of Thrombotic Thrombocytopenic Purpura (TTP) (2025), the recommended volume of plasma exchange per session is 2000–3000 mL. The first full-dose plasma exchange was performed on the 4th day after admission. Subsequently, due to insufficient inventory of AB-type plasma, the daily plasma exchange regimen was adjusted to 1000 mL of plasma plus 500 mL of 4% albumin. After 4 sessions of plasma exchange, the results of the externally submitted test were returned (see Table 1), which showed that the plasma ADAMTS13 activity was 0.62% and ADAMTS13 inhibitor was positive, confirming the diagnosis of immune-mediated thrombotic thrombocytopenic purpura (iTTP). At this stage, the patient’s platelet count (PLT) did not increase significantly, and the level of Lactate Dehydrogenase (LDH) still continued to rise, indicating no satisfactory therapeutic response (see Figure 2). It was considered that in addition to insufficient plasma exchange volume, the aggravated pulmonary infection was also a confounding factor. After escalating the antibiotic regimen to meropenem, the patient’s laboratory indicators gradually returned to normal. After 14 sessions of plasma exchange, the patient’s platelet count reached the normal range. After the platelet count remained normal for two consecutive measurements, plasma exchange was adjusted to an every-other-day regimen, and the patient still showed a favorable therapeutic response. Along with the improvement of laboratory indicators, the patient’s consciousness gradually recovered. Twenty-five days after admission, the patient was transferred from the intensive care unit (ICU) to the general ward and has remained in clinical remission to date.

## Discussion

Thrombotic thrombocytopenic purpura is a thrombotic microangiopathy that affects multiple organs and systems throughout the body. Its pathogenesis involves hereditary or acquired deficiency of von Willebrand factor cleaving protease, which leads to impaired cleavage of von Willebrand factor (vWF) multimers and subsequent microthrombosis. This process further causes ischemia, hypoxia and dysfunction of the involved organs, resulting in the corresponding clinical syndrome (1),The patient presents with abrupt onset of multiple conditions and is in a critical condition (2). Based on the distinct deficiency mechanisms of ADAMTS13, TTP is classified into iTTP and congenital TTP (cTTP). iTTP accounts for approximately 95% of all TTP cases, representing the most common clinical subtype, and it is frequently secondary to infections, medications, autoimmune diseases, malignancies, pregnancy and other underlying conditions (3). In the present case, after detailed inquiry into the patient’s medical history, no other family members presented similar symptoms, and no clear triggering the patient’s onset was identified. The patient was definitively diagnosed with iTTP based on the results of serum ADAMTS13 activity assay and ADAMTS13 inhibitory antibody testing.

Some patients with thrombotic thrombocytopenic purpura (TTP) may present with a “pentad” of clinical manifestations, including microangiopathic hemolytic anemia, thrombocytopenia, neuropsychiatric symptoms, fever, and renal involvement (4–6). However, the classic pentad is rare; approximately 60%–80% of cases manifest as a triad consisting primarily of microangiopathic hemolytic anemia, thrombocytopenia, and neuropsychiatric symptoms. Thus, this triad alone cannot serve as the sole diagnostic criterion (7).

ADAMTS13 enzyme activity is the only specific biomarker for TTP diagnosis, with plasma ADAMTS13 activity <10% representing a pathophysiological hallmark of TTP (8). The presence of ADAMTS13 inhibitors or positive IgG antibodies confirms a diagnosis of iTTP (9). Since ADAMTS13 enzyme level assessment requires time, clinical prediction models such as the Bentley score, French score, and PLASMIC score have been developed to aid in TTP diagnosis. Studies have shown that the PLASMIC score has a sensitivity of 81.7% and specificity of 71.4% for diagnosing TTP in high-risk patients (5). Additionally, incorporating the ratio of lactate dehydrogenase (LDH) level to the upper limit of normal (ULN) has been shown to enhance the diagnostic value of the PLASMIC score (10), enabling early treatment initiation prior to obtaining ADAMTS13 laboratory results. In contrast, the Bentley and French scores currently lack further clinical validation (11, 12). Clinicians must interpret and apply these scoring tools based on individual clinical contexts during the early diagnostic phase.

Therapeutic plasma exchange serves as the cornerstone of iTTP management, applicable to both confirmed cases and as the first-line emergency intervention for patients with high clinical suspicion. Current guidelines recommend the use of fresh frozen plasma (FFP), with a single exchange volume of 2000–3000 mL (13). Given that only approximately 4% of the population has blood group AB, this type of plasma is frequently in short supply. For therapeutic plasma exchange (TPE) in patients with blood group AB, group AB plasma free of hemolytic risk is the standard product for routine use, while transfusion of group A, B or O plasma confers a hemolytic risk. We proposed a TPE strategy combining albumin-plasma: albumin solution or a mixture of albumin and normal saline is administered during the initial phase of exchange, followed by plasma for the terminal exchange phase (14). This regimen removes ADAMTS13 inhibitors in the first half of the TPE procedure and replenishes functional ADAMTS13 protease in the latter half. When the albumin-plasma combination serves as the replacement fluid, multiple benefits are achieved. First, albumin maintains intravascular colloid osmotic pressure and hemodynamic stability. By replacing a portion of the exchange volume, it substantially cuts the total infusion volume of incompatible plasma, markedly reduces the risk of acute hemolysis induced by allogeneic blood group antibodies, and lowers the incidence of various transfusion-related adverse events such as hypersensitivity. Second, albumin alone lacks bioactive moieties including coagulation factors, immunoglobulins and complements; adjunctive low-volume plasma supplementation restores these essential components to avert bleeding tendency and immune dysregulation, providing concurrent coagulation and immune support. Third, albumin is readily available with abundant stockpiles. The combined approach mitigates the supply shortage of rare group AB plasma and ensures adequate, uninterrupted TPE for emergency resuscitation (15). Multiple studies have demonstrated that albumin-combined plasma exchange achieves platelet recovery in a shorter timeframe, is more cost-effective in resource utilization, and carries a lower risk of volume overload and cross-infection (15). Aggressive treatment with glucocorticoids or other immunosuppressive agents (e.g., rituximab) may help reduce the number of TPE sessions required for clinical recovery (16). Chander et al. found that caplacizumab in combination with glucocorticoids and rituximab could be an effective therapeutic option for iTTP patients who are unable or unwilling to undergo plasma exchange (17).

Clinical response is defined as a sustained platelet count ≥100 × 10^9^/L and lactate dehydrogenase (LDH) < 1.5 times the upper limit of normal (ULN) following treatments such as plasma exchange, with no new-onset or exacerbated ischemic organ damage. In clinical practice, patients exhibit significant heterogeneity in their responses to TPE. Our observations indicate that patients achieve clinical response more rapidly after the resolution of infectious factors (e.g., respiratory or urinary tract infections). This phenomenon may be attributed to the massive release of inflammatory mediators during infection, which increases vascular permeability and alters hemodynamics—thereby impairing blood flow and separation efficiency during TPE and reducing the clearance of pathogenic factors. Cui et al. demonstrated that infection control is a critical measure for managing infection-associated TTP, emphasizing that antibiotics should be administered as early as possible when clinically indicated to enhance therapeutic efficacy (18). We further found that the temporary use of broad-spectrum antibiotics in iTTP patients with concurrent infections can shorten the time to clinical response. For patients with refractory TTP, closer monitoring of relevant laboratory parameters, timely evaluation of treatment outcomes, and formulation of individualized therapeutic regimens are essential.

In conclusion, when encountering a patient presenting with neurological symptoms, multisystem impairment, and unexplained thrombocytopenia, microthrombotic disorders such as TTP should be excluded prior to platelet transfusion. Platelet transfusion in TTP patients is only indicated for life-threatening bleeding events (e.g., central nervous system hemorrhage). Once TTP is highly suspected or confirmed, TPE should be initiated promptly. Albumin-combined plasma exchange is an effective alternative when plasma supply is insufficient. For patients with refractory TTP, active identification and elimination of precipitating factors (e.g., infection) are critical. Rituximab and caplacizumab may be considered when necessary; however, their role as first-line therapies, as well as optimal dosage and timing, require validation through large-scale clinical trials.

## Data Availability

The original contributions presented in this study are included in this article/supplementary material, further inquiries can be directed to the corresponding author.
